# A rare case of gliclazide-induced symptomatic bradycardia in type 2 diabetes mellitus

**DOI:** 10.1210/jcemcr/luag161

**Published:** 2026-07-13

**Authors:** Amit Kumar, Sandeep Garg, Praveen Bharti, Bhvika Zutshi, Shweta Ambedare, Vedprakash Yadav

**Affiliations:** Department of Medicine, Maulana Azad Medical College and Lok Nayak Hospital, New Delhi 110002, India; Department of Medicine, Maulana Azad Medical College and Lok Nayak Hospital, New Delhi 110002, India; Department of Medicine, Maulana Azad Medical College and Lok Nayak Hospital, New Delhi 110002, India; Department of Medicine, Maulana Azad Medical College and Lok Nayak Hospital, New Delhi 110002, India; Department of Medicine, Maulana Azad Medical College and Lok Nayak Hospital, New Delhi 110002, India; Department of Medicine, Maulana Azad Medical College and Lok Nayak Hospital, New Delhi 110002, India

**Keywords:** type 2 diabetes, adverse drug reaction, sulfonylurea, bradycardia, gliclazide

## Abstract

Gliclazide, a second-generation sulfonylurea, is frequently utilized for glycemic management in type 2 diabetes mellitus (T2DM), attributed to its cardiovascular safety profile and minimal hypoglycemia risk. Sulfonylurea-induced bradycardia is a rare and underreported phenomenon. A 68-year-old male with a long history of T2DM and hypertension presented with presyncope, lethargy, and dizziness 10 days following the initiation of modified-release gliclazide at a dosage of 60 mg once daily. Upon evaluation, the heart rate was recorded at 42 beats per minute with a regular rhythm. Electrocardiogram indicated sinus bradycardia accompanied by intermittent junctional escape beats. Echocardiography, cardiac biomarkers, thyroid function tests, and electrolyte levels were within normal limits. Twenty-four-hour Holter monitoring revealed sinus bradycardia with pauses lasting up to 3.2 seconds. No evidence was found of underlying conduction abnormalities or concurrent use of other bradycardia-inducing medications. A diagnosis of bradycardia induced by gliclazide was established based on the temporal correlation and the resolution of symptoms following the discontinuation of the medication. After a modification in his antidiabetic treatment, the patient continued to exhibit no symptoms. This case underscores the necessity of identifying rare yet significant cardiac adverse effects associated with gliclazide, particularly in elderly patients, to facilitate prompt intervention and prevent unwarranted investigations.

## Introduction

Sulfonylureas have played a critical role in the management of type 2 diabetes mellitus (T2DM) for many years, especially in resource-limited settings where cost and accessibility are significant factors. Gliclazide, a second-generation sulfonylurea, is commonly used owing to its reduced risk of hypoglycemia, once-daily modified-release (MR) formulation, and a comparatively safer cardiovascular profile relative to earlier sulfonylureas like glibenclamide and tolbutamide [1, 2]. The ADVANCE trial established the safety and efficacy of gliclazide MR in reducing macrovascular and microvascular complications in individuals with T2DM [3].

Gliclazide functions by interacting with the sulfonylurea receptor 1 located on pancreatic β-cell K_ATP_ channels. This interaction results in channel closure, membrane depolarization, calcium influx, and subsequent insulin release [4]. ATP-sensitive potassium (K_ATP_) channels are expressed in cardiomyocytes, and their modulation influences cardiac repolarization and conduction [5]. Gliclazide is regarded as more selective for pancreatic tissue; however, potential off-target effects on cardiac conduction have been suggested.

Bradycardia is not commonly recognized as a side effect of gliclazide. The most frequently observed adverse effects are hypoglycemia, weight gain, and gastrointestinal disturbances [1]. Although infrequent cardiac events, such as bradyarrhythmia, have been reported with other sulfonylureas like glimepiride and glibenclamide [6, 7], specific reports linking gliclazide to these events are notably limited. The interaction of sulfonylureas with autonomic regulation and their potential to unmask underlying conduction system disease may be underestimated, especially in elderly patients or those on multiple medications [8].

We present a rare case of symptomatic sinus bradycardia induced by gliclazide, supported by temporal correlation, clinical resolution following discontinuation, and the exclusion of other common causes.

## Case presentation

A 68-year-old male with a decade-long history of T2DM and hypertension presented to the medicine outpatient department, reporting fatigue, generalized weakness, dizziness, and near-syncope episodes occurring over the past 10 days. The patient reported no history of chest pain, palpitations, dyspnea, fever, or recent infections. No history of trauma, alcohol use, or recent medication changes was noted, except for the introduction of a new oral antidiabetic medication. The patient had a history of T2DM diagnosed 10 years prior and hypertension diagnosed 8 years prior. There was no history of coronary artery disease, cardiac arrhythmias, or thyroid dysfunction. The patient also had no prior history of sulfonylurea use.

The patient was taking metformin 500 mg twice daily, telmisartan 40 mg once daily, and amlodipine 5 mg once daily. The family physician initiated gliclazide MR 60 mg once daily 2 weeks prior to the onset of symptoms to enhance glycemic control. Glycated hemoglobin (HbA1c) was 8% (SI: 64 mmol/mol) (reference range, ≤ 5.7%, [SI: 39 mmol/mol]) at the time of start of gliclazide MR and gliclazide MR was started due to its cost-effectiveness.

## Diagnostic assessment

On examination, vital signs were SpO_2_ 98% on room air, respiratory rate 16/minute, blood pressure 130/70 mmHg, and heart rate 42 beats per minute (bpm) with regular rhythm. On general examination, there was no cyanosis, edema, or pallor. On cardiovascular examination, there were no gallop or murmurs; only sinus bradycardia was noted. On neurological examination, the patient was alert, oriented, and had no focal deficits, and other systems were unremarkable.

An electrocardiogram (ECG) was done and revealed sinus bradycardia (heart rate 40 bpm), occasional junctional escape beats, and normal PR and QT intervals. A previous ECG from 1 year earlier showed a normal sinus rhythm with a heart rate of 84 bpm. Complete blood count showed hemoglobin level of 13.2 g/dL (SI: 132 g/L) (reference range, 13-17 g/dL [SI: 130-170 g/L]), white blood cells were 7600/µL (SI: 7.6 4-11 × 10^9^/L) (reference range, 4000-11000/µL [SI: 4-11 × 10^9^/L]), and platelet counts were 2.25 lac/µL (SI: 225 × 10^9^/L) (reference range, 1.5-4.5 lac/µL [SI: 150-440 × 10^9^/L]). His random blood glucose was 112 mg/dL (SI: 6.2 mmol/L) (reference range, 70-140 mg/dL [SI: 3.9-7.8 mmol/L]), serum sodium level was 142 mEq/L (SI: 142 mmol/L) (reference range, 135-145 mEq/L [SI: 135-145 mmol/L]), serum potassium level was 4.3 mEq/L (SI: 4.3 mmol/L) (reference range, 3.5-5.0 mEq/L [SI: 3.5-5.0 mmol/L]), serum total calcium level was 9.2 mg/dL (SI: 2.3 mmol/L) (reference range, 8.6-10.2 mg/dL [SI: 2.1-2.6 mmol/L]), serum magnesium level was 1.9 mg/dL (SI: 0.78 mmol/L) (reference range, 1.7-2.4 mg/dL [SI: 0.7-1.0 mmol/L]) and thyroid stimulating hormone (TSH) level was 2.1 µIU/mL (SI: 2.1 mIU/L) (reference range, 0.4-4.0 µIU/mL [SI: 0 .4-4.0 mIU/L]). Kidney function test showed blood urea 20 mg/dL (SI: 7.1 mmol/L) (reference range, 15-45 mg/dL [SI: 2.5-7.8 mmol/L]), serum creatinine was 0.7 mg/dL (SI: 62 µmol/L) (reference range, 0.7-1.2 mg/dL [SI: 60-110 µmol/L]), and liver function tests were as follows: total bilirubin 0.5 mg/dL (SI: 8.6 µmol/L) (reference range, 0.5-1.2 mg/dL [SI: 5-21 µmol/L]), direct bilirubin 0.1 mg/dL (SI: 1.7 µmol/L) (reference range, 0-0.3 mg/dL [SI: 0-5 µmol/L]), aspartate aminotransferase 34 U/L (SI: 34 U/L) (reference range, 10-40 U/L [SI: 10-40 U/L]), alanine aminotransferase 23 U/L (SI: 23 U/L) (reference range, 7-56 U/L [SI: 7-56 U/L]), alkaline phosphatase 67 U/L (SI: 67 U/L) (reference range, 40-140 U/L [SI: 40-140 U/L]), total protein 7.3 g/dL (SI: 73 g/L) (reference range, 6-8 g/dL [SI: 60-80 g/L]), and albumin 3.9 g/dL (SI: 39 g/L) (reference range, 3.5-5.0 g/dL [SI: 35-50 g/L]) as summarized in Table 1. High-sensitivity troponin-I was negative.

**Table 1:** Key laboratory findings of the patient

Test	Conventional units (reference range)	SI units (reference range)
Hemoglobin	13.2 g/dL (13-17 g/dL)	132 g/L (130- 170 g/L)
White blood cells	7600/µL (4000-11000/µL)	7.6 × 10^9^/L (4-11 × 10^9^/L)
Platelets count	2.25 lac/µL (1.5-4.5 lac/µL)	225 × 10^9^/L (150-440 × 10^9^/L)
Random blood glucose	112 mg/dL (70-140 mg/dL)	6.2 mmol/L (3.9-7.8 mmol/L)
Serum sodium	142 mEq/L (135-145 mEq/L)	142 mmol/L (135-145 mmol/L)
Serum potassium	4.3 mEq/L (3.5-5.0 mEq/L)	4.3 mmol/L (3.5-5.0 mmol/L)
Total calcium levels	9.2 mg/dL (8.6-10.2 mg/dL)	2.3 mmol/L (2.1-2.6 mmol/L)
Magnesium levels	1.9 mg/dL (1.7-2.4 mg/dL)	0.78 mmol/L (0.7-1.0 mmol/L)
TSH	2.1 µIU/mL (0.4-4.0 µIU/mL	2.1 mIU/L (0 .4-4.0 mIU/L)
Blood urea	20 mg/dL (15-45 mg/dL)	7.1 mmol/L (2.5-7.8 mmol/L)
Creatinine	0.7 mg/dL (0.7-1.2 mg/dL)	62 µmol/L (60-110 µmol/L)
Total bilirubin	0.5 mg/dL (0.5-1.2 mg/dL)	8.6 µmol/L (5-21 µmol/L)
Direct bilirubin	0.1 mg/dL (0-0.3 mg/dL)	1.7 µmol/L (0-5 µmol/L)
AST	34 U/L (10-40 U/L)	34 U/L (10-40 U/L)
ALT	23 U/L (7-56 U/L)	23 U/L (7-56 U/L)
ALP	67 U/L (40-140 U/L)	67 U/L (40-140 U/L)
Total protein	7.3 g/dL (6-8 g/dL)	73 g/L (SI: 60-80 g/L)
Serum albumin	3.9 g/dL (3.5-5.0 g/dL)	39 g/L (35-50 g/L)

Abbreviations: ALP, alkaline phosphatase; ALT, alanine aminotransferase; AST, aspartate aminotransferase; TSH, thyroid-stimulating hormone.

Echocardiogram revealed normal left ventricular systolic function with an ejection fraction of approximately 60%. There were no regional wall motion abnormalities or structural or valvular abnormalities observed. Twenty-four-hour Holter monitoring showed a minimum heart rate of 36 beats per minute; the mean heart rate was 44 beats per minute. Findings indicated multiple sinus pauses, with the longest duration recorded at 3.2 seconds. There was no evidence of atrioventricular block, and occasional junctional escape rhythms were observed. There was no significant correlation observed with sleep or the postprandial period.

## Treatment

Considering the temporal relationship with the initiation of gliclazide MR, the lack of other medications associated with bradycardia (such as beta-blockers and digoxin), and the exclusion of structural or metabolic causes, gliclazide-induced bradycardia was hypothesized. The Naranjo adverse drug reaction probability score was 5, signifying a “probable” drug-related adverse event.

Gliclazide was promptly discontinued and was not reintroduced. Other medications, such as metformin and antihypertensives, were maintained without alteration.

## Outcome and follow-up

The patient was monitored in the hospital for 48 hours. On day 2, the heart rate increased to 58 bpm, and the patient was asymptomatic. A subsequent ECG demonstrated sinus rhythm at a rate of 64 bpm. After one week of follow-up, Holter monitoring indicated normalization of heart rate (average heart rate 68 bpm), with no further pauses or arrhythmias recorded. The patient was transitioned to a dipeptidyl peptidase-4 inhibitor, vildagliptin, for glycemic management, with no recurrence of bradycardia observed over the subsequent three months.

## Discussion

Bradycardia is characterized by a heart rate of less than 60 beats per minute, although it can be a normal finding in well-trained individuals. Symptomatic pathological bradycardia necessitates assessment for structural, metabolic, and pharmacological etiologies. The lack of electrolyte abnormalities, hypothyroidism, structural heart disease, and other bradycardia-inducing agents suggests that gliclazide is the probable cause.

The pharmacological profile of gliclazide encompasses its mechanism of action, pharmacokinetics, and therapeutic applications in the management of diabetes mellitus. Gliclazide primarily functions as a sulfonylurea, stimulating insulin secretion from pancreatic beta cells. Its pharmacokinetic properties include oral bioavailability, peak plasma concentration, and elimination half-life, which are critical for understanding its clinical effects.

Gliclazide, a second-generation sulfonylurea, interacts with the sulfonylurea receptor 1 subunit of K_ATP_ channels in pancreatic β-cells, thereby facilitating insulin secretion [4]. Gliclazide exhibits greater selectivity for pancreatic tissue compared to glibenclamide or glimepiride, resulting in a reduced risk of hypoglycemia and off-target effects [1]. K_ATP_ channels are present in cardiomyocytes, especially in the conduction system, and their modulation can influence cardiac excitability and automaticity [5].

Gliclazide-induced bradycardia, while exceedingly uncommon, may be explained by several proposed mechanisms are modulation of cardiac K_ATP_ channels: sulfonylureas may inhibit cardiac K_ATP_ channels, influencing repolarization and potentially leading to bradyarrhythmias. Gliclazide, while relatively selective, may still demonstrate partial activity on myocardial K_ATP_ channels, especially in predisposed individuals [9]. Regarding utonomic imbalance, gliclazide may indirectly enhance parasympathetic tone or diminish sympathetic drive, which could lead to sinus bradycardia. Research indicates that sulfonylureas can induce autonomic modulation in specific animal models [10]. Regarding subclinical hypoglycemia, the patient in this case did not exhibit documented hypoglycemia; however, asymptomatic episodes may have elevated vagal tone, potentially leading to bradyarrhythmia. Nonetheless, this remains conjectural, given that glucose levels were consistently normal during the monitoring period. Synergistic effects with other medications: the patient was administered amlodipine and telmisartan, neither of which is commonly associated with bradycardia. Pharmacodynamic interactions, particularly in elderly patients, may reveal subclinical conduction defects.

Gliclazide-induced bradycardia is not extensively documented; however, other sulfonylureas have been associated with this condition. Bradycardia induced by glimepiride was observed in an elderly diabetic patient and resolved following discontinuation of the medication [11]. Singh et al reported a case of glibenclamide-associated sinus bradycardia in a diabetic patient who exhibited significant bradycardia without other identifiable causes [7]. Sulfonylureas do not correlate with an elevated risk of all-cause mortality, cardiovascular mortality, myocardial infarction, or stroke. Current research supports the safety of sulfonylureas; an absolute risk of 0.5% may be definitively dismissed [12]. The case reports and pharmacodynamic insights provide evidence for the likelihood of gliclazide-induced bradyarrhythmia, especially in elderly patients or those with undetected sinoatrial node dysfunction. This case underscores a frequently overlooked adverse effect associated with a widely used antidiabetic medication. The increasing utilization of gliclazide MR among older adults, due to its favorable safety profile, necessitates that clinicians remain vigilant for cardiac conduction abnormalities, particularly in patients who exhibit fatigue or dizziness soon after the initiation of the medication.

Although gliclazide is usually regarded as safe and cardioneutral, infrequent side effects like bradycardia may occur, particularly in older or predisposed individuals. ECG monitoring, thorough history taking, and a high index of suspicion can all aid in the early detection and management of such events.

## Learning points

- Gliclazide-induced bradycardia, though extremely rare, should be considered when unexplained bradycardia occurs after starting therapy.
- Prompt recognition and drug discontinuation lead to complete recovery and help avoid unnecessary cardiac evaluations.
- Clinician vigilance is essential, especially in elderly patients, as even drugs with good cardiovascular safety profiles can cause uncommon adverse effects.

## Contributors

A.K. was primarily involved in the clinical evaluation, diagnosis, and management of the patient and drafted the initial manuscript. V.Y. participated in inpatient management, monitoring of hypoglycemic episodes, and collection of clinical and biochemical data. P.B. contributed to diagnostic evaluation, exclusion of alternative causes of hypoglycemia, and interpretation of laboratory findings. B.Z. was involved in patient care, follow-up assessment, and interpretation of treatment response following withdrawal of gliclazide. S.A. contributed to the review of literature regarding sulfonylurea-induced hypoglycemia and assisted in clinical correlation of the case findings. S.G. supervised the overall clinical management of the patient, contributed to diagnostic and therapeutic decision-making, and critically reviewed the manuscript for important intellectual content. All authors were directly involved in patient care and approved the final manuscript for publication.

## Data Availability

Data sharing is not applicable to this article as no datasets were generated or analyzed during the current study.
